# Effect of type 2 diabetes, surgical incision, and volatile anesthesia on hemodynamics in the rat

**DOI:** 10.14814/phy2.13352

**Published:** 2017-07-17

**Authors:** Carol T. Bussey, Regis R. Lamberts

**Affiliations:** ^1^ Department of Physiology – HeartOtago School of Biomedical Sciences University of Otago Dunedin New Zealand

**Keywords:** Diabetes mellitus, hemodynamics, surgery, type 2

## Abstract

Diabetic patients have increased cardiac complications during surgery, possibly due to impaired autonomic regulation. Anesthesia lowers blood pressure and heart rate (HR), whereas surgical intervention has opposing effects. The interaction of anesthesia and surgical intervention on hemodynamics in diabetes is unknown, despite being a potential perioperative risk factor. We aimed to determine the effect of diabetes on the integrative interaction between hemodynamics, anesthesia, and surgical incision. Zucker type 2 diabetic rats (DM) and their nondiabetic littermates (ND) were implanted with an intravenous port for drug delivery, and a radiotelemeter to measure mean arterial blood pressure (MAP) and derive HR (total *n* = 50). Hemodynamic pharmacological responses were assessed under conscious, isoflurane anesthesia (~2–2.5%), and anesthesia–surgical conditions; the latter performed as a laparotomy. MAP was not different between groups under conscious conditions (ND 120 ± 6 vs. DM 131 ± 4 mmHg, *P > *0.05). Anesthesia reduced MAP, but not differently in DM (ND −30 ± 6 vs. DM −38 ± 4 ΔmmHg, *P > *0.05). Despite adequate anesthesia, surgical incision increased MAP, which tended to be less in DM (ND +21 ± 4 vs. DM +13 ± 2 ΔmmHg, *P = *0.052). Anesthesia disrupted central baroreflex HR responses to sympathetic activation (sodium nitroprusside 10 *μ*g·kg^−1^, ND conscious 83 ± 13 vs. anesthetized 16 ± 5 Δbpm; *P *<* *0.05) or to sympathetic withdrawal (phenylephrine 10 *μ*g·kg^−1^, ND conscious −168 ± 37 vs. anesthetized −20 ± 6 Δbpm; *P *<* *0.05) with no additional changes observed after surgical incision or during diabetes. During perioperative conditions, type 2 diabetes did not impact on short‐term hemodynamic regulation. Anesthesia had the largest hemodynamic impact, whereas surgical effects were limited to modulation of baseline blood pressure.

## Introduction

Regulation of hemodynamics is normally maintained by autonomic control through a balance between the sympathetic and parasympathetic nervous systems. This tight balance is important for adequate perfusion of organs at rest to maintain homeostasis, and is vital during acute periods of increased metabolic demand. Cardiovascular autonomic function is acutely challenged in the perioperative setting (Vinik and Ziegler 2007; Oakley and Emond 2011) by both anesthesia and surgical intervention. For instance, anesthesia is well known to lower blood pressure and heart rate (HR) (Altholtz et al. 2006), and to impair baroreflex responsiveness (Yoshimoto et al. 2011). In contrast, surgical interventions are known to increase blood pressure and HR (Abraham et al. 1981; Gemes et al. 2009; Charlet et al. 2011; Yeh et al. 2012).

The growing population with type 2 diabetes impacts heavily on cardiovascular health, and it is well known that the long‐term impact of diabetes is associated with autonomic dysregulation of hemodynamics (Vinik and Ziegler 2007). An often overlooked, but clinically important, cardiovascular consequence is that patients with diabetes have increased requirements for surgical treatments. Following surgery, patients with diabetes need longer hospital stays and have poorer survival compared to patients without diabetes (Alserius et al. 2009). Moreover, patients with diabetes are subject to a higher incidence of perioperative hemodynamic complications, even for noncardiac‐related surgeries (Knuttgen et al. 1990; Vohra et al. 1993; Vinik and Ziegler 2007). The lowering effects of anesthesia on blood pressure and HR are augmented during diabetes (Amour et al. 2004; Crespo et al. 2011; Oakley and Emond 2011). Recently, we showed that long‐term metabolic adaptations, associated with type 2 diabetes and obesity, altered the *α*‐ and *β*‐adrenergic function, and its acute interaction with isoflurane anesthesia (Bussey et al. 2014b). However, the interaction of anesthesia with a surgical intervention during the perioperative setting in type 2 diabetes is unknown and might have a significant impact on blood pressure and HR regulation.

Therefore, we aimed in this study to determine the effect of type 2 diabetes on the interaction between hemodynamics, anesthesia, and surgical incision. Because we recently showed in rats in vivo that type 2 diabetes impaired *β*‐adrenergic function during volatile anesthesia (Bussey et al. 2014b), we hypothesized that a surgical incision (laparotomy) on top of anesthesia would further impair blood pressure and HR regulation in the rats with diabetes. To address this we used our recently developed approach that allows determination of how a surgical incision (under anesthesia) affects mean arterial blood pressure (MAP) and HR regulation compared to anesthesia alone and conscious conditions (Bussey et al. 2014a, 2014b). Type 2 diabetic (Zucker Diabetic Fatty [ZDF]) rats and their nondiabetic littermates were implanted with a vascular access port and a radiotelemetric transmitter to inject intravenous drugs and to measure in vivo abdominal aortic blood pressure, respectively. Hemodynamic pharmacological responses were assessed under conscious, isoflurane anesthesia, and anesthesia–surgical conditions.

## Methods

### Animals

All procedures were approved by the University of Otago Animal Ethics Committee and were conducted in accordance with the New Zealand Animal Welfare Act (1999).

**Figure 1:**
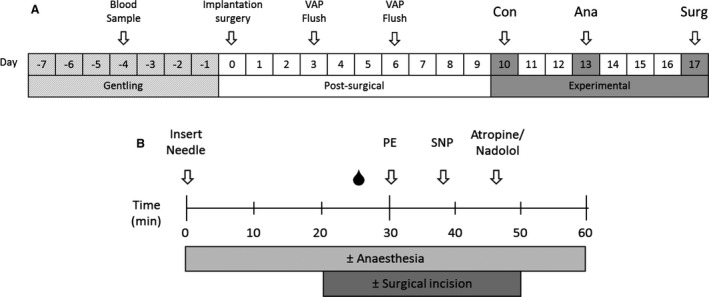
Experimental design. The overall experimental design (A). Animals were gentled for 7 days prior to implantation surgery of a radiotelemeter and vascular access port (VAP), followed by 10‐day postsurgical recovery during which VAP patency was maintained by biweekly flush. Here, after the 10‐day experimental period was started, consisting of three measurement sessions: under conscious conditions, isoflurane anesthesia, and isoflurane anesthesia including a sham surgery. To prevent adrenergic receptor desensitization at least a 2‐day period was maintained between sessions. A measurement session of the experimental protocol (B). An equilibration period of 20 min followed insertion of the needle into the VAP. After 30 min, or 10 min after surgical incision, phenylephrine (PE,* α*‐adrenoceptor agonist, 10 *μ*g·kg^−1^) and sodium nitroprusside (SNP, nitric oxide donor, 10 *μ*g·kg^−1^) were injected 8 min apart in random order, with the VAP flushed between each administration. At the end, because of their longer lasting effects, the response to atropine (muscarinic receptor blocker, 1 mg·kg^−1^) or nadolol (nonselective *β*‐adrenoceptor blocker, 4 mg·kg^−1^) was tested.

ZDF rats (DM) are derived from a selected subset of the Zucker strain, which spontaneously develop diabetes from 12 weeks of age due to impaired pancreatic beta‐cell function (Paulsen et al. 2010). This strain is a well‐accepted model of obese type 2 diabetes mellitus, with their lean littermates used as in‐strain nondiabetic controls (ND). Male rats (*N* = 50; University of Otago breeding facility with stock from Charles River Laboratories, Wilmington, MA) were housed at 20 ± 1°C under a 12‐h light–dark cycle and provided with food and water ad libitum. All ZDF animals were maintained on Purina 5008 diet (LabDiet^®^, St Louis, MO) as recommended by the supplier. Animals were gentled daily for 1 week prior to surgery (Fig. 1A). Plasma samples were collected via the tail vein following an 8‐h fast 4 days prior to surgery. Plasma glucose concentrations were determined using a glucometer (Roche, Basel, Switzerland), and insulin was measured by ELISA (Millipore, Billerica, MA).

### Surgical procedures

Dual implantation of a vascular access port (VAP) and a radiotelemeter was performed on 20‐week‐old animals under isoflurane anesthesia (2–2.5% in 100% oxygen 1 L·min^−1^; Minrad Inc., Bethlehem, PA) as described previously (Bussey et al. 2014a, 2014b) with strict adherence to aseptic procedures. Analgesia (carprofen 5 mg kg^−1^; Norbrook, Newry, Northern Ireland) and antibiotic (trimethoprim and sulphamethazine 30 mg·kg^−1^; Virbac, Carros, France) were administered subcutaneously. Vascular Access Ports (VAP™; ROP‐3H, hydromer‐coated polyurethane, 3Fr; Access Technologies, Skokie, IL) were primed with heparin sodium (100 IU·mL^−1^; Hospira Australia, Mulgrave, Australia). The VAP reservoir was secured on the back of the animal between the scapulae with the VAP cannula tunneled subcutaneously to the femoral vein. A radiotelemeter with pressure‐sensitive tip (TRM54P; Telemetry Research, Millar Instruments, Houston, TX) was implanted into the abdominal aorta. Animals were allowed a 10‐day postsurgical recovery period before experimentation commenced (Fig. 1A). Ten days after surgery, animals had normal food and water intake, gained normal body weight, displayed normal mobility, and presented no pain behavior (writhing, back arch, stagger, belly press, or tremor), which is in agreement with a previous study in rats also observing normal mobility, body temperature, and absence of local hyperexcitability at the scar area 10 days postsurgery (Charlet et al. 2011). Surgical failure, postoperative complications, and instrument failure prevented 18% of animals from completing this study. The VAP was flushed with 0.4 mL heparin sodium (100 IU·mL^−1^) at minimum twice weekly to maintain patency.

### Experimental Procedures

Experiments were performed twice weekly to reduce stress to the animals, as well as ensuring complete drug clearance and avoiding potential desensitization of adrenoceptors. This 2–3 days allowance between experimental sessions, along with the 10‐day postsurgical recovery period, also served to minimize potential effects of repeated exposure to volatile anesthetics, generally described as lasting 24–72 h (Lucchinetti et al. 2007). VAPs were accessed under strict aseptic conditions using a Huber point needle (PG24‐625; Access Technologies, Skokie, IL), following application of a short‐acting local analgesic (5% lidocaine/prilocaine; AstraZeneca, North Ryde, NSW, Australia).

During an experimental session (Fig. 1B), hemodynamic measures were equilibrated for 20 min following restraint and needle insertion, with subsequent stress‐free injection of pharmacological substances testing the autonomic regulation under three different conditions: conscious (Con), anesthesia (Ane), and anesthesia–surgery (Surg). For measures under anesthetized conditions, induction was undertaken with isoflurane (5%), and maintenance under slightly variable percentages to ensure proper anesthetic depth for the full 60 min (ND Ane 2.2 ± 0.1% vs. DM Ane 2.3 ± 0.1%; ND Surg 2.4 ± 0.1% vs. DM Surg 2.7 ± 0.1% isoflurane, *P *<* *0.05 DM Surg vs. all other groups). Adequate depth of anesthesia was assessed regularly during the 60 min via lack of withdrawal reflex due to toe pinch of rear paw, the gold standard in rodent surgery (National Research Council (US) Committee for the Update of the Guide for the Care and Use of Laboratory Animals 2011). The anesthesia–surgery session was similar to the anesthesia conditions, with the addition of a 3‐cm full thickness lateral abdominal opening. This was performed by stepwise incision of the skin through the muscle layers and into the abdomen maintained for 30 min. At the completion of all experimental sessions, animals were euthanized via pentobarbital overdose. A subset of animals, both ND and DM, underwent a set of experiments with repeated conscious conditions and no exposure to anesthesia or surgery, as a technical and time control. There were no significant differences in any of the measures across these experiments (*n* = 5–7, data not shown).

The pharmacological substances tested were *α*‐adrenergic agonist phenylephrine (PE, 10 *μ*g·kg^−1^) and the nitric oxide donor sodium nitroprusside (SNP; 10 *μ*g·kg^−1^), which were randomized and administered at 8‐min intervals with a following saline flush (Fig. 1B). Here after, because of the drugs longer lasting effects, each animal was assigned to receive either atropine (muscarinic receptor blocker, 1 mg·kg^−1^) or nadolol (nonselective *β*‐adrenoceptor blocker, 4 mg·kg^−1^) for all three experimental sessions. Any access via the VAP was concluded by injection of 0.4 mL heparin sodium (100 IU·mL^−1^) to prevent coagulation. All chemicals were from Sigma‐Aldrich (St. Louis, MO) and diluted in saline (0.9% NaCl; Baxter, Toongabbie, Australia) unless otherwise stated.

### Data and statistical analyses

Blood pressure data were derived from the telemeter according to the manufacturer's instructions, and acquired using LabChart^®^ 7 software (ADInstruments, Dunedin, New Zealand). HR and MAP were derived from blood pressure recordings using the LabChart^®^ blood pressure module and averaged over every 10 consecutive cycles. Hemodynamic responses were assessed as the calculated change between the peak response to and the baseline immediately preceding each individual bolus injection.

Statistical analysis was performed for baseline characteristics via *t*‐test or Mann–Whitney rank sums test where the assumptions were not met, or via two‐way repeated measures ANOVA for all hemodynamic data. Differences between groups were identified using Student–Newman–Keuls post hoc analysis (Sigmaplot™ 12.0, Systat Software Inc., Chicago, IL) and significance assumed at the level of *P *<* *0.05. Data are expressed as mean ± standard error of the mean (SEM).

## Results

### Animal characteristics

Type 2 diabetic rats (DM) exhibited body weights 25% higher than their nondiabetic littermates (ND), with no signs of cardiac hypertrophy (Table 1). This was accompanied by significantly greater abdominal adiposity, as indicated by higher epididymal fat pad weight. Type 2 diabetic animals also exhibited hyperglycemia and hyperinsulinemia, both characteristics of their diabetic condition.

**Table 1:** Baseline animal characteristics

	ND	DM
Body weight (g)	333 ± 6	417 ± 14^a^
Epididymal fat weight (g)	1.5 ± 0.2	6.4 ± 0.7^a^
Epididymal fat weight/tibia length (g·cm^−1^)	0.4 ± 0.1	1.9 ± 0.2^a^
Heart weight (g)	1.49 ± 0.06	1.52 ± 0.06
Heart weight/tibia length (g·cm^−1^)	0.41 ± 0.02	0.44 ± 0.02
Fasting plasma glucose (mmol·L^−1^)	6.4 ± 0.4	19.5 ± 4.4^a^
Fasting plasma insulin (ng·mL^−1^)	1.2 ± 0.3	8.0 ± 2.2^a^

Baseline characteristics of 20‐week‐old Zucker type 2 ds (ZDF, DM) and their nondiabetic littermates (ND).

a Significantly different from control littermates, *n* = 14 per group; *P* < 0.05, values are means ± SE.

### Baseline hemodynamics

**Figure 2:**
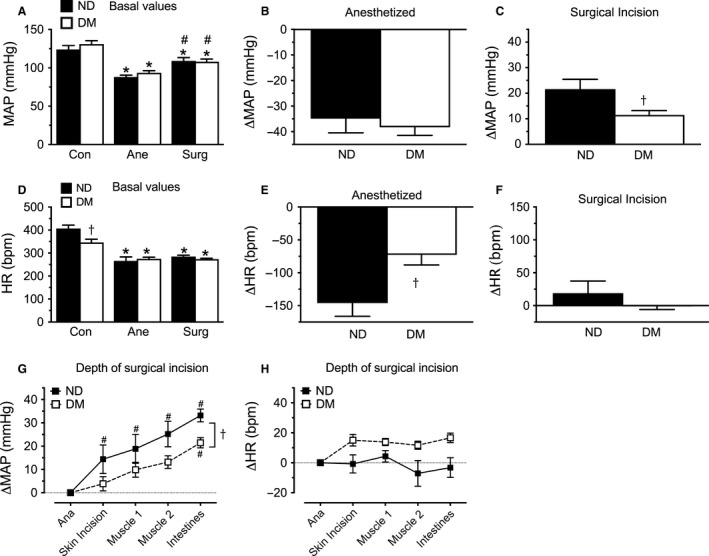
Baseline hemodynamics. Under conscious (Con) conditions mean arterial pressure (MAP) was not different (A) and heart rate (HR) was lower (D) in diabetic (DM) compared to nondiabetic (ND) animals. MAP was reduced by isoflurane anesthesia (Ana), along with HR in both ND and DM animals. The decrease in MAP to anesthesia (B) was similar, but the decrease in HR (E) was less in DM rats. Surgical incision (Surg) increased MAP (C), which was slightly less in DM animals, but surgical incision did not change HR (F). Detailed hemodynamic effects of surgical incision showed that MAP (G) rose under isoflurane anesthesia with increasing depth of surgical incision from skin through the muscle layers into the abdomen, with no change in HR (H). The increase in MAP was slightly less in the DM compared to ND animals (A–F) *n* = 11–14 per group, (G–H) *n* = 6 per group; *P <* *0.05 vs. Con, ^#^ *P *<* *0.05 vs. Ana, ^†^ *P *<* *0.05 vs. ND.

Baseline hemodynamics were assessed under conscious resting conditions (Con), following stabilization of isoflurane anesthesia (Ane), and during surgical incision under isoflurane anesthesia (Surg) (Fig. 2). MAP was not different between diabetic and nondiabetic animals under conscious conditions (MAP: ND 124 ± 6 vs. DM 131 ± 5 mmHg, *P *>* *0.05, Fig. 2A), whereas HR was lower in diabetic animals (HR: ND 407 ± 17 vs. DM 346 ± 16 bpm, *P < *0.05, Fig. 2D). Isoflurane anesthesia significantly reduced both MAP and HR in all animals, eliminating differences in HR. The decrease in MAP following isoflurane anesthesia was not different between groups (MAP: ND −35 ± 6 vs. DM −38 ± 3 ΔmmHg, *P *>* *0.05; Fig. 2B), whereas the decrease in HR was less in the diabetic animals (HR: ND −146 ± 20 vs. DM −73 ± 15 Δbpm, both *P < *0.05; Fig. 2E). Surgical incision increased MAP, an effect that was lessened in diabetic rats (ND +22 ± 4 vs. DM +11 ± 2 ΔmmHg, *P < *0.05; Fig. 2C), but surgical incision did not significantly change HR (ND +19 ± 18 vs. DM −0.3 ± 6 ΔHR, *P > *0.05; Fig. 2F). More detailed hemodynamic analysis during the surgical period revealed that MAP (Fig. 2G), but not HR (Fig. 2H), rose with increasing depth of surgical incision from skin through the muscle layers into the abdomen. This occurred despite adequate anesthesia, as indicated by lack of change in MAP or HR in response to a toe pinch (data not shown).

### Parasympathetic system

**Figure 3:**
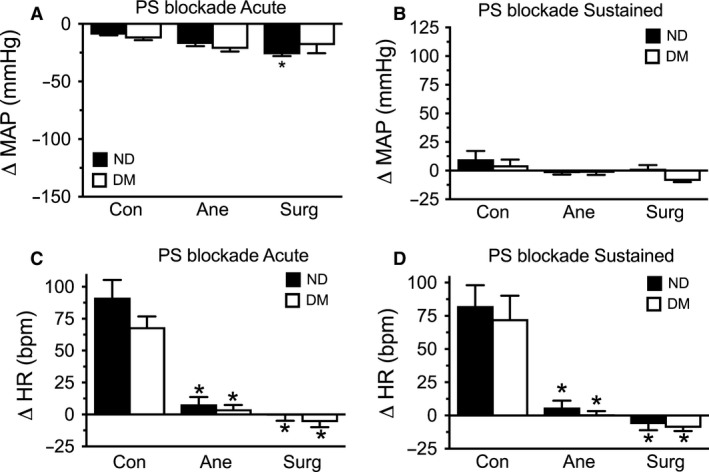
Parasympathetic system. Parasympathetic (PS) blockade with atropine 1 mg·kg^−1^ immediately, within seconds, tended to reduce mean arterial pressure (MAP) (A), which was not sustained after 10 min (B). Surgical incision (Surg), but not anesthesia (Ane), significantly exacerbated the acute vasodilation in response to PS blockade in the nondiabetic (ND) animals. This was accompanied by a marked acute (C) and sustained (D) increase in HR in the conscious (Con) animals, which was abolished under anesthesia with no additional effect of surgical incision. No differences were observed between ND and diabetic (DM) animals. *n* = 5–8, *P <* *0.05 vs. Con.

To determine whether anesthesia and surgical incision affected the parasympathetic regulation of MAP and HR, the effect of atropine, a muscarinic receptor blocker, was investigated. Surprisingly, atropine administration elicited an acute decrease in MAP (Fig. 3A), which was not sustained after 10 min (Fig. 3B). Surgical incision, but not anesthesia, significantly exacerbated this peak reduction in MAP, occurring seconds after the parasympathetic blockade, although no differences were observed between diabetic and nondiabetic animals under any of the three conditions (Fig. 3A and B). This was accompanied by a marked acute and sustained increase in HR (Fig. 3C and D) in the conscious animals, however, this tachycardia was completely abolished by anesthesia with no additional effect of surgical incision or diabetes.

### *β*‐adrenergic system

**Figure 4:**
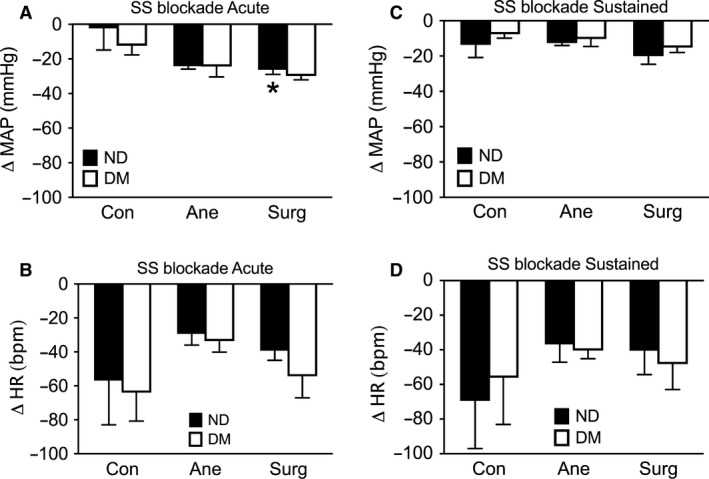
*β*‐adrenergic system. Blockade of the sympathetic system (SS) with nadolol 4 mg·kg^−1^ immediately (within seconds) tended to reduce mean arterial pressure (MAP) (A), although this effect did not persist 10 min after administration (B). Surgical incision (Surg), but not anesthesia (Ane), significantly exacerbated the acute vasodilation in response to SS blockade in nondiabetic (ND) animals. SS blockade with nadolol induced an acute (C) and sustained (D) decrease in heart rate (HR) in the conscious (Con) animals, which was not significantly altered by anesthesia or surgical incision. No differences were observed between ND and diabetic (DM) animals. *n* = 5–6, *P <* *0.05 vs. Con.

To determine whether anesthesia and surgical incision affected the *β*‐adrenergic regulation of MAP and HR, the effect of nadolol, a nonselective *β*‐adrenoceptor blocker, was investigated. Nadolol administration acutely tended to reduce MAP (Fig. 4A), although this effect did not persist 10 min after administration (Fig. 4B). Surgical incision, but not anesthesia, significantly exacerbated the peak reduction in MAP, occurring seconds after the *β*‐adrenergic blockade, with no differences observed between diabetic and nondiabetic animals under any of the three conditions (Fig. 4A and B). As expected, *β*‐adrenergic blockade with nadolol induced a significant and sustained decrease in HR (Fig. 4C and D) in the conscious animals. This bradycardia was not significantly affected by anesthesia, surgical incision, or diabetes.

### Baroreflex responses

**Figure 5:**
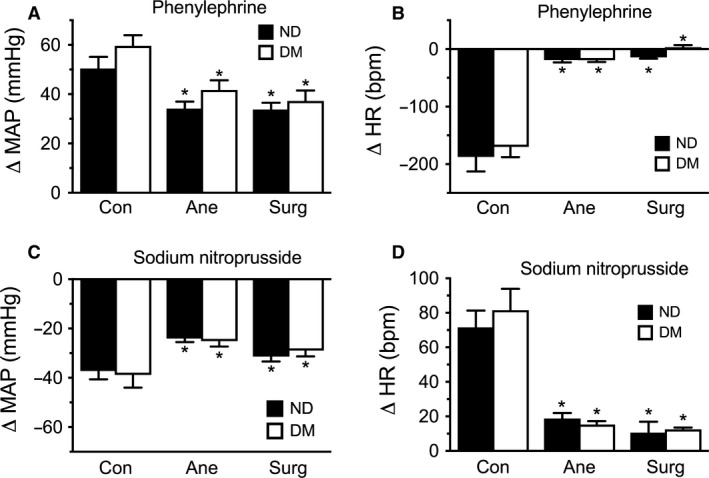
Baroreflex responses. Phenylephrine (10 *μ*g·kg^−1^) increased mean arterial pressure (MAP) (A) and lowered heart rate (HR) (B) in conscious (Con) conditions. Anesthesia (Ane) reduced the peripheral vasoconstriction and disrupted the central sympathetic withdrawal baroreflex, with no additional effect of surgical incision (Surg). Sodium nitroprusside (10 *μ*g·kg^−1^) decreased MAP (C) and increased HR (D), whereas anesthesia reduced the peripheral vasodilatation and disrupted the central sympathetic activation baroreflex with no additional effect of surgical incision. No differences were observed between nondiabetic (ND) and diabetic (DM) animals for any of the responses. *n* = 11–14, *P <* *0.05 vs. Con.

Finally, the effect of anesthesia and surgical incision on the complex baroreflex responses was determined. Administration of a therapeutic dose of phenylephrine (PE), an *α*‐adrenoceptor agonist (Overgaard and Dzavik 2008), primarily elicited a rapid, transient increase in MAP in conscious rats (Fig. 5A). Under anesthesia, the increase in MAP to *α*‐adrenoceptor stimulation was reduced, with surgical incision during anesthesia producing a similarly reduced increase in MAP compared to the conscious animals. No significant differences were observed between diabetic and nondiabetic animals under any of the three conditions. Secondary to the changes in MAP, PE elicited a reduction in HR (Fig. 5B). This baroreflex‐mediated bradycardia was almost completely abolished by anesthesia, despite the pressor stimulus being only mildly reduced, with no additional effect of surgical incision (Fig. 5B). Moreover, the PE‐induced reduction in HR, a measure of central sympathetic withdrawal and parasympathetic activation, was not different between diabetic and nondiabetic animals under any of the three conditions.

Administration of sodium nitroprusside, (SNP), a nitric oxide donor, primarily elicited a rapid, transient decrease in MAP in conscious rats (Fig. 5C). This vasodilator‐induced decrease in MAP was reduced under anesthesia, an effect that was not significantly altered by surgical incision (Fig. 5C). Secondary to the changes in MAP, SNP elicited an increase in HR (Fig. 5D). This baroreflex‐mediated tachycardia was markedly reduced under anesthesia, despite only slight reduction in the vasodilatory response, with no additional effect by surgical incision (Fig. 5D). The SNP‐induced decreases in MAP and increases in HR, a measure of central sympathetic activation and parasympathetic withdrawal, were not different between diabetic and nondiabetic animals under any of the three conditions.

## Discussion

We aimed to determine the effect of type 2 diabetes on the integrative interaction between hemodynamics, anesthesia, and surgical incision. We assessed MAP and HR in nondiabetic and diabetic ZDF rats in vivo during baseline and pharmacological responses targeting autonomic cardiovascular control. We found that type 2 diabetes showed no interaction with anesthesia, only a slightly reduced increase in blood pressure during the surgical intervention. Isoflurane anesthesia markedly impaired blood pressure regulation with a loss of baroreflex responses and disrupted (para) sympathetic control. Surgical incision increased baseline blood pressure, increased acute sensitivity of blood pressure regulation during (para) sympathetic blockade, but had no impact on baroreflex or (para) sympathetic heart rate control.

We had expected that the presence of uncontrolled diabetes would markedly affect HR and blood pressure regulation, especially under anesthetic and surgical intervention. The ZDF animals displayed the characteristics of an uncontrolled type 2 diabetic animal model with hyperglycemia, hyperinsulinemia, and obesity due to increased adiposity. Under conscious baseline conditions, the type 2 diabetic animals were normotensive with a markedly lower HR, which is in agreement with previous studies (Marsh et al. 2007; Radovits et al. 2009; Bussey et al. 2014b; Thaung et al. 2015). The lower HR in the diabetic animals relates to the intrinsic properties of the pacemaker cells in the sinoatrial node as the difference is also observed in isolated heart preparations without autonomic control (Thaung et al. 2015). However, diabetes elicited only a slightly reduced increase in blood pressure during the surgical incision compared to nondiabetic animals. Counterintuitively, this would suggest impaired vasoconstriction in diabetes, however, most likely the systemic vasculature in our diabetic ZDF rats was already in a more constrictive state. This is supported by the results that MAP of the diabetic ZDF rats was similar to their nondiabetic littermates, but with a reduced HR (and slightly lower stroke volume; Thaung et al. 2015), suggesting that total peripheral resistance is increased in the diabetic rats. Furthermore, we did not observe any major influences of diabetes during any of our other hemodynamic challenges, which suggests that isoflurane anesthesia might have the most impact of the three provided hemodynamic challenges. Moreover, our animal model does not have other comorbidities (e.g., hypertension), which often occur in patients with diabetes and could have exacerbated the hemodynamic changes.

The surgical incision (laparotomy) in our study raised basal MAP, but not HR, with a larger rise in MAP at increasing depth of surgical incision. This increase in blood pressure occurred despite adequate anesthesia as indicated by lack of change in HR or withdrawal reflex due to toe pinch. Increases in MAP due to surgical intervention are well known (Abraham et al. 1981; Gemes et al. 2009; Charlet et al. 2011; Yeh et al. 2012). Previous reports indicate it could result from a pain‐induced increase in central sympathetic drive, causing increased systemic levels of vasoactive catecholamines (Desborough 2000), however, these studies always reported an associated increase in HR (Charlet et al. 2011; Yeh et al. 2012). Yeh et al. (2012), who performed their experiments under “light” anesthetic conditions to deliberately increase sympathetic drive during the surgical incision, showed that sedation with the *α*_2_‐adrenergic agonist dexmedetomidine was able to prevent the increases in MAP and HR. Moreover, Charlet et al. (2011), with the use of analgesic ropivacaine, also showed that the increase in HR does not relate to systemic effects. Second, a drop in systemic blood volume due to the surgical incision could have also caused a lower blood pressure. However, this would result in a baroreflex‐mediated increase in HR, which again we did not observe. Nevertheless, blood flow may still be redistributed within the different organ systems, equating to no overall change, which is supported by studies in rats (Yeh et al. 2012), but conflicted by several studies in pigs (Schwarz et al. 2001; Sack et al. 2002; Hiltebrand et al. 2011). This finding might have been confounded by the slight variation in isoflurane percentages needed to maintain surgical levels of anesthesia across groups; although the lack of response differentiation between nondiabetic and diabetic animals suggests this is unlikely. The lack of a change in HR due to the laparotomy suggests that most likely local vasoconstriction mechanisms of resistance vessels in the abdomen (e.g., splanchnic and mesenteric arteries) are responsible for the increase in blood pressure. Alternatively, surgical intervention can cause release of cytokines (Desborough 2000) or alter the immune response and coagulation (Collins et al. 1977; Levi and van der Poll 2010).

An additional interesting finding is that during the acute, first few seconds of (para) sympathetic blockade a larger drop in MAP (but not HR) was observed during surgical incision compared to the conscious and anesthetic conditions. Vasodilatory effects of atropine have previously been described at greater doses than required to produce tachycardia, and linked to interference with peripheral *α*‐adrenergic signaling (Abraham et al. 1981; Shinoura et al. 2002). Therefore, the increased sensitivity of blood pressure regulation with acute (para) sympathetic blockade during surgical intervention may relate to differential activation of local vasoconstrictive pathways during the hypertensive response to surgical incision.

None of the other interventions, neither the baroreflex tests nor the sustained (para) sympathetic blockade, changed any of the hemodynamic parameters during surgical intervention; most likely due to the fact that isoflurane anesthesia already had completely disrupted the autonomic regulation of these integrative hemodynamic systems. The significant reduction in both HR and MAP, the reduced peripheral vasoreactivity, and the disrupted central withdrawal and activation baroreflexes by isoflurane anesthesia confirmed several earlier studies describing the hypotensive and cardiodepressant effects of volatile anesthetics (Graves et al. 1974; Housmans and Murat 1988; Lynch and Frazer 1989; Nakao et al. 1989; Bernard et al. 1990; McKinney et al. 1993; Malan et al. 1995). These dramatic disruptive effects of volatile anesthesia on hemodynamics are well illustrated by our results that during SNP injection under isoflurane anesthesia MAP dropped (Fig. 5C), however there was a disproportional lack of HR augmentation (Fig. 5D). To exclude the hemodynamic effects of anesthesia an additional group with surgical stress alone (without anesthesia) would be required, however, this is not feasible from animal welfare point of view. Second, it could be argued that the severity of our laparotomy (3 cm for 30 min) was not sufficient to observe any additional hemodynamic effects compared to more severe cardiothoracic or orthopedic interventions. Under these circumstances, it has been observed that patients with diabetes have increased postoperative mortality and a higher incidence of postoperative cardiac events (Axelrod et al. 2002; Carson et al. 2002; Noordzij et al. 2007; Halkos et al. 2010; Castelvecchio et al. 2011). More importantly, our approach to compare conscious hemodynamic responses, with the responses during anesthesia with/without surgery, does mimic the clinical perioperative settings of many smaller surgical interventions. Thus, from the results of our experiments, performed under our relatively “mild” surgical conditions, we conclude that anesthesia is the major modulator of the adverse hemodynamic changes during the perioperative period.

In conclusion, during perioperative conditions type 2 diabetes did not impact on short‐term hemodynamic regulation. Anesthesia had the largest hemodynamic impact under our conditions, whereas a surgical intervention only had minor additional effects. Thus, while it is important to consider the impact of all manipulations for perioperative management, the effect of diabetes and surgery appears to be minimal.

## Conflict of Interest

None declared.
